# Clinical and Psychosocial Predisposing Factors Associated With Anaesthesia Failure During Non‐Surgical Root Canal Treatment: A National Dental Practice‐Based Research Network Study

**DOI:** 10.1111/iej.70171

**Published:** 2026-05-18

**Authors:** Linda J. Liu, Ronald Ordinola‐Zapata, W. Craig Noblett, Ellen Funkhouser, Alan S. Law, Donald R. Nixdorf, Ernest W. N. Lam, Rahma Mungia, Gregg H. Gilbert, The National Dental Practice‐Based Research Network Collaborative Group

**Affiliations:** ^1^ Division of Endodontics, Department of Restorative Sciences, School of Dentistry University of Minnesota Minneapolis Minnesota USA; ^2^ Division of Preventive Medicine, School of Medicine University of Alabama at Birmingham Birmingham Alabama USA; ^3^ Specialty Practices West Roseville Minnesota USA; ^4^ Division of TMD and Orofacial Pain University of Minnesota Minneapolis Minnesota USA; ^5^ Faculty of Dentistry University of Toronto Toronto Ontario Canada; ^6^ Department of Periodontics, School of Dentistry The University of Texas Health San Antonio San Antonio Texas USA; ^7^ Department of Clinical and Community Sciences, School of Dentistry University of Alabama at Birmingham Birmingham Alabama USA

**Keywords:** endodontics, local anaesthesia, practice‐based research network

## Abstract

**Aim:**

This study aimed to identify dentist, patient, and pre‐treatment clinical characteristics associated with local anaesthesia failure during non‐surgical endodontic treatment.

**Methodology:**

Data were collected from the National Dental Practice‐Based Research Network study entitled “Predicting Outcomes of Root Canal Treatment (PREDICT)”, which included 1723 patients. Local anaesthesia failure during treatment was defined as patient‐reported pain of 3 or greater on a 0‐to‐10‐point scale. Pre‐treatment factors included patient demographics, psychosocial constructs, and pre‐treatment clinical findings (e.g., abnormal sensitivity to cold, biting, percussion, palpation). Characteristics with *p* < 0.1 (after adjustment for clustering with generalized estimating equations) were entered into a model to identify independent associations with failed anaesthesia, and odds ratios were calculated to measure the strength of these associations.

**Results:**

A total of 16% of patients reported intra‐operative local anaesthesia failure. Failure was associated with patient age < 55 years (*p* = 0.05), dental treatment fear (“some” to “extremely afraid”) (*p* = 0.005), mandibular teeth (*p* = 0.005), and greater numbers of abnormal pre‐treatment diagnostic findings (*p* = 0.02). Protective factors included having treatment done by an endodontist (*p* = 0.002), and the patient being non‐Hispanic White (*p* = 0.007). Comparisons of local anaesthetic techniques by dentist type (general dentist vs. endodontist) showed statistically significant differences in the bivariate analysis, but none remained significant after adjustment for clustering of patients within dentist.

**Conclusions:**

Local anaesthesia failed in 16% of cases. Pre‐treatment fear was the only significant psychosocial predictor of intra‐operative pain. Failure was more likely in mandibular teeth and when multiple abnormal tests were present pre‐operatively (e.g., cold sensitivity, percussion tenderness). Younger patients reported more pain, and endodontists had lower pain rates than general dentists.

## 1 Introduction

Safe and effective local anaesthesia is essential in ensuring patient comfort during endodontic treatment. Despite advancements in anaesthetic agents and techniques, local anaesthesia failure remains a significant clinical challenge (Hargreaves and Keiser 2002). Inadequate anaesthesia can cause pain and reduce patient trust in dental care, negatively affecting treatment and future oral health habits. Understanding the multifactorial nature of these failures is important for improving patient care in endodontics.

The causes of local anaesthesia failure may be multifactorial (Hargreaves and Keiser 2002; Nusstein et al. 2010), including variables such as patient anatomical complexity, the technical aspects of local anaesthesia delivery, and the presence of symptomatic irreversible pulpitis (Hargreaves and Keiser 2002; Kanaa et al. 2006; Nusstein et al. 2010). The underlying mechanisms include local inflammation, which increases peripheral sensitization and lowers the patient's pain threshold, as well as central sensitization driven by prolonged nociceptive input, both of which could reduce the efficacy of local anaesthetics (Byers and Närhi 1999; Hargreaves and Keiser 2002; Henry and Hargreaves 2007; Obadah et al. 2026).

Effectiveness studies are uncommon in endodontic research. Research conducted in routine clinical settings offers broader applicability compared to efficacy studies. On the other hand, the lack of standardized protocols in effectiveness models can introduce variability that may limit internal validity (Singal et al. 2014). Prior analyses of an earlier National Dental Practice‐Based Research Network dataset (Network study 17) identified factors associated with local anaesthesia failure during RCT, observing higher failure rates when treatment was provided by general dentists and among patients reporting diabetes or preoperative tooth pain, and lower failure rates when treated by endodontists or when a sinus tract was present (Weitz et al. 2021).

Pre‐treatment psychosocial factors are clinically relevant and dental fear has been linked to increased pain perception during procedures (Armfield 2010; Armfield and Heaton 2013); catastrophizing amplifies pain reporting (Bartley and Fillingim 2013; Haythornthwaite et al. 2024); and depression and perceived stress are associated with lower pain tolerance and increased analgesic use (Michaelides and Zis 2019). To date, there are limited data in the literature that comprehensively evaluate psychosocial and clinical pre‐treatment factors as predictors of intraoperative pain. This investigation uses data from a cohort Predicting Outcomes of Root Canal Treatment (PREDICT) designed to capture patient‐reported pain during and after RCT. The aim was to determine whether pre‐treatment clinical and psychosocial factors independently predict intraoperative anaesthesia failure during RCT (primary aim). We hypothesized that pre‐treatment psychosocial factors, in addition to established clinical factors such as mandibular tooth location and multiple abnormal diagnostic findings, would predict intraoperative anaesthesia failure.

## 2 Materials and Methods

The PREDICT study (National Dental PBRN 2017) was granted human research ethics approval by the University of Alabama at Birmingham (Protocol X161121003), the University of Minnesota (Protocol RNI00002290), and the University of Toronto (Protocol 34 105).

This prospective observational study was conducted from April 6, 2017 to November 30, 2017 (6‐month enrollment period). It follows the Preferred Reporting items for Observational studies in Endodontics (PROBE) 2023 reporting guidelines. To assess local anaesthesia failures and associated variables, clinical and radiographic data from patients were obtained through the Network's 153 participating dentists, including 104 general dentists and 49 endodontists (Mungia et al. 2025a, 2025b). The study consecutively enrolled participants over 6 months, collecting electronic data from patients and dentists before and after RCT (See Figure S1). Results related to severe pre‐operative pain in patients receiving RCT, and demographic characteristics have been recently reported (Mungia et al. 2025a, 2025b). Consistent with previous Network research (Gilbert et al. 2022; Cunha‐Cruz et al. 2023; Thyvalikakath et al. 2022), the full study protocol, dentist recruitment flyer, and all study data forms utilized in this study are available to the public (Recruiting for Studies: Ongoing, Upcoming & Completed | National Dental PBRN) (National Dental PBRN 2017).

### 2.1 Sample Size

In the parent effectiveness study (National Dental PBRN, Mungia et al. 2025a, 2025b), 1704 patients contributed data. Using the rule‐of‐thumb that ~15 outcome events are needed per predictor in the final multivariable logistic regression model, this anaesthesia failure secondary analysis has sufficient events to support at least 10 predictors in the final model.

### 2.2 Patient Eligibility

#### 2.2.1 Inclusion Criteria

Patients were eligible if they were 18 or 19 years old or older, depending on state requirements, could return to the clinic after 12 months, were willing to provide contact information for another person, had internet access, gave informed consent, could read in English or Spanish, and had a permanent tooth requiring RCT.

#### 2.2.2 Exclusion Criteria

Patients were excluded if they had a previous RCT on the study tooth, required RCT on more than one tooth simultaneously, or did not complete the procedure. Radiographs were taken and assessed by the treating dentist.

##### 2.2.2.1 Patient‐Reported Characteristics

The pre‐RCT patient questionnaire collected information on pain experienced immediately before and during the 7 days prior to RCT (Von Korff et al. 1992; Law et al. 2015), as well as medications used for dental pain in that same period. Patients underwent screening for temporomandibular disorders (TMD) through a questionnaire comprising six standardized items (Fonseca Alonso et al. 2017; Daline et al. 2023; Gonzalez et al. 2011) that addressed symptoms experienced in the 30 days preceding RCT. TMD was assessed because patients with TMD frequently present with myofascial pain, more‐severe endodontic symptoms, and more psychological factors (Daline et al. 2023).

Data for six psycho‐social measures were collected using a 4‐ or 5‐point Likert scale: (1) treatment outcome expectation (Nixdorf et al. 2016; Bartley and Fillingim 2013); (2) dental treatment fear (Milgrom et al. 1988); (3) pain catastrophizing (Sullivan et al. 1995); (4) depression using the PHQ‐2 questionnaire (Kroenke et al. 2003; Seo and Park 2015); (5) anxiety using the GAD‐2 questionnaire (Spitzer et al. 2006; Schalet et al. 2014); and (6) stress using the Perceived Stress Scale (Cohen et al. 1983; Ingram et al. 2016) (see National Dental PBRN website for coding of variables). Pre‐RCT pain assessment was conducted based on reports of current pain immediately preceding the RCT, as well as the most‐severe pain experienced during the 7 days prior to the RCT. Each pain measure was rated on a zero to 10 scale, where zero was ‘no pain,’ and 10 was ‘pain as bad as it could be’ (Von Korff et al. 1992; Nixdorf et al. 2016). The post‐RCT patient questionnaire asked about pain during RCT on a zero to 10 scale, as well as demographics.

##### 2.2.2.2 Clinical Findings Reported by the Dentist

Before treatment, dentists completed a questionnaire that recorded information such as tooth number, pulpal and clinical findings (including sensitivity to cold, percussion, biting pressure, and palpation), probing depths, whether the tooth was an abutment to a fixed or removable partial denture, the existence of proximal contacts, swelling, presence of a draining sinus tract, mobility, and any periapical radiolucency (Mungia et al. 2025b, 2025a). Dentists filled out a revised version of the American Association of Endodontists (AAE) Case Difficulty Assessment Form, which included a list of nine possible factors that could make the procedure challenging: (1) limited ability to open mouth; (2) gag reflex; (3) full coverage restoration present; (4) calcification present within the pulp; (5) longest cusp tip to apex distance was > 21 mm; (6) curvature was substantial or S‐shaped; (7) whether each root had 1 additional canal that was not clearly visible; (8) if there was incomplete root development; and (9) if root resorption was present.

##### 2.2.2.3 Defining Anaesthesia Failure and Local Anaesthesia Techniques

Anaesthesia failure was defined as patient‐reported pain of 3 or greater on a zero to 10 scale. The dentist questionnaire completed following the RCT elicited information on the different anaesthetic techniques used. One question asked “Were any of the following necessary to obtain adequate anesthesia to perform treatment? (Mark all that apply): (a) Second injection of the same type into the same location; (b) Second injection of the same type into a slightly different location; (c) Block anesthesia technique different from the one previously provided; (d) Periodontal ligament injection; (e) Intraosseous injection other than the periodontal ligament injection; (f) Intrapulpal injection”. When entered into the database, all results were entered as either Yes (1) or No (0). All data were collected electronically.

##### 2.2.2.4 Statistical Analysis

Characteristics assessed for association with failed anaesthesia were from 6 groups: (1) dentist characteristics; (2) patient demographics; (3) pre‐RCT pain and TMD; (4) medications; (5) psychosocial constructs; and (6) pre‐RCT clinical findings.

All statistical analyses were performed using the SAS 9.4 (Copyright 2025 SAS Institute Inc.) software. Descriptive statistics, such as mean (SD) and median (IQR), were used to summarize each characteristic of interest (listed above). The relationship between each characteristic and unsuccessful anaesthesia was determined by dichotomizing psychosocial variables at the threshold that yielded the highest Chi‐square statistic with pre‐RCT pain. This allowed for easier comparison of associations with failed anaesthesia, since most were dichotomous. In a univariable fashion, each characteristic was entered into a logistic regression model that used a generalized estimating equations (GEE) method that adjusted for clustering of patients within the practice, implemented using PROC GENMOD in SAS with CORR = EXCH option. Analyses were performed within each domain (viz., dentist characteristics, patient demographics, medications, psycho‐social measures, and dentist pre‐RCT assessment) to determine which variables‐ and, when appropriate, composite measures‐ would be included in the final model. Characteristics with *p*‐values less than 0.1 (after adjustment for clustering with GEE) were included into a full model to determine independent associations with failed anaesthesia (analyses within domains are presented in a Table S1). No overall stepwise elimination, namely, backwards, because of concerns of “over inflating” statistical significance of finding (Olusegun et al. 2015; Heinze and Dunkler 2017). A pseudo‐*R* ^2^ was computed as a measure of variance in failed anaesthesia that was explained by the model. Odds ratios (OR) were computed to quantify the strength of associations. All *p*‐values reported below and in the accompanying tables regarding failed anaesthesia have been adjusted to account for patient clustering within individual dentists.

## 3 Results

### 3.1 Study Population

A total of 153 Network dentists screened, confirmed eligibility for, and obtained consent from 1860 patients, as shown in Figure S1. Of the 137 patients not enrolled: 68 were deemed ineligible by dentists; 6 were disqualified due to relocation or lack of email; contact was lost with 3; and 60 withdrew without completing baseline questionnaires. Additionally, pain data were unavailable for 2 of the final 1723 patients, and the dentist did not finish the pre‐RCT questionnaire for 14 patients. Therefore, the following final data analyses were performed on a total of 1704 patients.

### 3.2 Dentist Characteristics

Among the 153 dentists surveyed, the mean age was 51.3 years (SD = 11.7), with a median of 52 and an interquartile range (IQR) from 42 to 62. Most participants were male (73%), non‐Hispanic White (70%), private practice owners (74%)—over half of whom operated solo practices—and general dentists (68%). The average number of patients enrolled per dentist was 11.2 (SD = 10.4), with a median of 8 patients (IQR: 4 to 50) and a range from 1 to 50. Endodontists and general dentists were comparable in age, race, and ethnicity; however, endodontists enrolled more than twice as many patients as general dentists (mean [SD], 19.0 [13.7] vs. 7.6 [5.6], *p* < 0.001) and were less likely to be owners of solo practices (21% vs. 52%, *p* < 0.001).

Incidence of local anaesthesia failure when pain level was ≥ 3 on a zero to 10 scale during RCT was reported by 274 patients (16% of the eligible study population). Regarding the association of dentist characteristics with patients experiencing failed anaesthesia, patients of dentists 65 years of age or older were more likely to report pain during RCT, although this was not statistically significant (OR = 1.45, *p* = 0.12). Patients of endodontists were less likely to report pain during RCT (OR = 0.61, *p* = 0.001), compared to general dentists.

### 3.3 Patient Characteristics

Patient characteristics are summarized in Table 1. The mean age of the patients was 48.3 years (SD = 15.8, range: 18 to 94, media*n* = 49, IQR: 36 to 69). Most were female (59%), and 69% were non‐Hispanic White. Also, a majority (76%) had some form of dental insurance, and almost half (46%) had completed a bachelor's degree or higher. Half of the patients had used over‐the‐counter (OTC) pain medication for their symptoms during the 7 days prior to RCT. Systemic antibiotics were prescribed to 28% of patients by the dentist, and 19% had been prescribed pain medication. Herbal remedies were used by 5% of patients. Almost half of the patients (48%) reported that the worst pain they had endured during the 7 days prior to RCT was severe (≥ 7 on a scale from zero to 10), and 21% reported that their current (immediately prior to RCT) pain was severe. In total, 41% of patients screened positive for TMD. Most patients (75%) expected a “very good treatment outcome,” while 34% were “somewhat to extremely afraid”. Stress of > 8 (on a scale from 2 to 10) was reported by 55% of patients, while 38% reported > 3 anxiety (on a 2 to 10 scale). Depression > 3 (on a 2 to 10 scale) was reported in 28% of patients, and 34% reported pain catastrophizing > 5.

**TABLE 1:** Characteristics of 1704 patients who received root canal treatment (RCT) overall, and by whether the patient reported pain during the RCT of 3 or greater (on a zero [no pain] to 10 [as bad as can be] scale).

Characteristic	All		Pain ≥ 3 during RCT		*p* ^b^	Odds ratio^c^
	*N* = 1704					
	*N*	%	*N*	Row %^a^		
*Patient demographics*						
Gender					0.9	0.98
Female	1004	59%	159	16%		
Male (referent)	694	41%	115	17%		
Missing *n* = 6						
Age (years)					< 0.001	1.75
Less than 55 years	1034	61%	195	19%		
55 or older (referent)	648	39%	73	11%		
Missing *n* = 22						
Non‐Hispanic White race‐ethnicity					< 0.001	0.55
No	516	31%	114	22%		
Yes	1151	69%	153	13%		
Missing *n* = 37						
Patient highest level of formal education					0.4	1.13
Less than Bachelor's degree	903	54%	153	17%		
Bachelor's degree or higher (referent)	762	46%	112	15%		
Missing *n* = 39						
Whether the patient has any dental insurance					0.048	1.34
No	415	24%	56	13%		
Yes	1289	76%	218	17%		
Missing *n* = 0						
*Pain sensitivity*						
Current pre‐RCT pain is severe					0.006	1.58
No (zero to 6)	1339	79%	196	15%		
Yes (7 to 10)	364	21%	78	21%		
Missing *n* = 1						
Worst pain 7 days prior RCT is severe					0.002	1.54
No (zero to 6)	892	52%	119	13%		
Yes (7 to 10)	809	48%	155	19%		
Missing *n* = 3						
Screen positive for temporomandibular disorders					0.2	1.26
No	1004	59%	148	15%		
Yes	700	41%	126	18%		
Missing *n* = 0						

Medications for tooth patient took during the 7 days prior to RCT						
Any pain medications for tooth					0.01	1.44
No	719	42%	96	13%		
Yes	982	58%	178	18%		
Missing *n* = 3						
Antibiotics from dentist					0.9	0.99
No	1231	72%	199	16%		
Yes	470	28%	75	16%		
Missing *n* = 3						

Psychosocial measures						
RCT outcome expectation (on 1–4 scale)					0.2	0.82
Expect poor to good (1 to 3)	425	25%	78	18%		
Expect very good RCT outcome	1277	75%	196	15%		
Missing *n* = 2						
Dental treatment fear (on 1–5 scale)					< 0.001	1.62
Not at all or a little afraid (1 or 2)	1131	66%	155	14%		
Somewhat to extremely afraid (3 to 5)	572	34%	119	21%		
Missing *n* = 1						
Pain catastrophizing (on 2–10 scale)					0.4	1.13
Scores 2 to 4	1106	66%	172	16%		
Scores of 5 or higher	565	34%	98	17%		
Missing *n* = 33						
Anxiety score (on 2–8 scale)					0.01	1.35
Score of 2	1041	62%	148	14%		
Scores 3 or higher	636	38%	121	19%		
Missing *n* = 27						
Depression (on 2–8 scale)					0.09	1.30
Scores of 2	1217	72%	182	15%		
Scores of 3 or higher	471	28%	89	19%		
Missing *n* = 16						
Stress score (on 4–20 scale)					0.002	1.52
Scores 4 to 7	750	45%	98	12%		
Scores of 8 or higher	914	55%	171	19%		
Missing *n* = 40						

^a^ *p*‐value adjusted for clustering of patients within dentist using generalized estimating equations (GEE).

^b^ Row %: Denominator is *N* under “All” column.

^c^ Odds ratio: Referent group is “No” if a response option, lower categories for psychosocial variables. For demographics, indicated with referent in applicable category.

Regarding the association of patient characteristics with failed anaesthesia, patients who were less than 55 years of age were more likely to report pain during RCT (OR = 1.75, *p* < 0.001), while non‐Hispanic White patients were less likely (OR = 0.55, *p* < 0.001). Patients with dental insurance were more likely to report pain during RCT (OR = 1.34, *p* = 0.05); however, when these three patient demographics were considered jointly in one model, only age, race, and ethnicity were statistically significant and entered in the full model described below. Patients who had taken pain medications (OTC or prescription) for their symptoms were more likely to report pain during RCT (OR = 1.44, *p* = 0.01). There was no association between a positive screening result for TMD and patients reporting pain during RCT. Both pain measures (current pre‐RCT pain is severe, and worst pain 7 days prior RCT is severe) were associated with patients reporting pain during RCT. When assessed together in one model, only “worst pain being severe” retained an association of *p* < 0.1. Most of the psychosocial measures were associated with patients reporting pain during RCT at a *p* = 0.1, viz., treatment fear (OR = 1.62, *p* < 0.001), stress (OR = 1.52, *p* = 0.002), anxiety (OR = 1.38, *p* = 0.01) and depression (OR = 1.30, *p* = 0.09).

### 3.4 Pre‐RCT Clinical Findings

Results representing the clinical findings reported by the dentist are summarized in Table 2. Anterior teeth accounted for 12% of the study population, premolars for 27%, and molars for 60%. Root canal treatments were performed on mandibular teeth in 47% of cases. Diagnostic results indicated that 69% of teeth were tender to percussion, 65% exhibited tenderness to biting pressure, 53% demonstrated abnormal responses to cold, and 29% were tender to palpation; a variable indicating the number of findings was defined, specifically has values 0, 1, 2, or 3. The most common reasons dentists anticipated that the RCT would be difficult were the presence of pulpal calcifications (25%), long cusp‐to‐tip apex distance (24%), the presence of a full coverage coronal restoration (24%), a patient with limited mouth opening (15%), not all root canals visible (13%), and substantial curvature or S‐shaped canals (11%).

**TABLE 2:** Dentist characteristics and clinical information reported by the dentist pre‐Root Canal Treatment (RCT), overall and by whether the patient reported pain during RCT of 3 or greater (on a zero [no pain] to 10 [as bad as can be] scale).

Characteristic	All		Pain ≥ 3 during RCT		*p* ^b^	Odds ratio^c^
	*N* = 1704					
	*N*	%	*N*	Row %^a^		
*Dentist characteristics*						
Endodontist					0.004	0.61
No (General Dentist)	781	46%	155	20%		
Yes	923	54%	119	13%		
Missing *n* = 0						
Dentist age: 65 years or older					0.12	1.45
No	1511	91%	233	15%		
Yes	155	9%	34	22%		
Missing *n* = 38						
*Pre‐RCT data reported by dentist*						
Molar (tooth type)					0.2	1.23
No (anterior or premolar)	682	40%	103	15%		
Yes	1022	60%	171	17%		
Missing *n* = 0						
Mandibular (tooth position)					0.01	1.38
No (maxillary)	904	53%	126	14%		
Yes	800	47%	148	19%		
Missing *n* = 0						
Diagnostic findings (Responds/tender to cold)					0.3	1.16
No	790	46%	121	15%		
Yes	910	54%	152	17%		
Missing *n* = 4						
Percussion					0.006	1.57
No	521	31%	62	12%		
Yes	1174	69%	210	18%		
Missing *n* = 9						
Biting pressure					< 0.001	1.68
No	600	35%	72	12%		
Yes	1096	65%	202	18%		
Missing *n* = 8						
Palpation					0.02	1.44
No	1195	71%	172	14%		
Yes	499	29%	101	20%		
Missing *n* = 10						
Any diagnostic finding					0.03	1.71
No	177	10%	19	11%		
Yes	1526	90%	255	17%		
Missing *n* = 1						
Number of diagnostic findings					< 0.001	1.29
0	177	10%	19	11%		
1	312	18%	29	9%		
2	425	25%	76	18%		
3	639	38%	116	18%		
4	150	9%	34	23%		
Missing *n* = 1						
*Other clinical characteristics*						
Any proximal contact					0.2	0.67
No	66	4%	15	23%		
Yes	1635	96%	259	16%		
Missing *n* = 3						
Exhibits radiolucency					0.4	1.12
No	933	55%	142	15%		
Yes	758	45%	130	17%		
Missing *n* = 13						
Any movement					0.2	1.22
No	1288	76%	202	16%		
Yes	403	24%	72	18%		
Missing *n* = 13						
Probing depth ≥ 5 mm					0.12	0.76
No	1504	89%	246	16%		
Yes	177	11%	25	14%		
Missing *n* = 23						
Swelling present					0.07	1.47
No	1518	90%	233	15%		
Yes	165	10%	37	22%		
Missing *n* = 21						
Draining sinus tract					0.3	0.74
No	1572	93%	255	16%		
Yes	121	7%	16	13%		
Missing *n* = 11						
Tooth abutment to removable or fixed partial denture					0.2	0.72
No	1581	94%	259	16%		
Yes	109	6%	13	12%		
Missing *n* = 14						
*Reasons procedure difficult* ^*d*^						
Calcifications present within pulp					0.9	0.99
No	1286	75%	210	16%		
Yes	418	25%	64	15%		
Longest > 21 mm cusp tip to apex					0.6	1.10
No	1292	76%	202	16%		
Yes	412	24%	72	17%		
Crown restoration present					0.3	0.84
No	1304	77%	219	17%		
Yes	400	23%	55	14%		
Patient limited ability open mouth					0.5	1.13
No	1453	85%	228	16%		
Yes	251	15%	46	18%		
Not all root canals clearly visible					0.8	1.07
No	1475	87%	236	16%		
Yes	229	13%	38	17%		
Curvature substantial or S‐shaped					0.095	1.38
No	1514	89%	235	16%		
Yes	190	11%	39	21%		
Patient gag reflex					0.4	1.41
No	1637	96%	259	16%		
Yes	67	4%	15	22%		
Root resorption					0.6	1.27
No	1660	97%	265	16%		
Yes	44	3%	9	20%		

^a^ *p*: *p*‐value adjusted for clustering of patients within dentist using generalized estimating equations (GEE).

^b^ Row %: Denominator is *N* under all column.

^c^ Odds ratio: Referent group is “No”.

^d^ There were none missing as the question was “check all that apply.” There wasn't a “no” option which is needed to ascertain number missing. Also, “Incomplete root development” as an option under possible difficulties only present with an *n* = 8; too rare to assess.

Mandibular teeth, the number of abnormal diagnostic findings, and the presence of swelling were the only clinical findings associated with pain during RCT (at *p* < 0.1). When considered together, only a tooth being in the mandible and the number of abnormal diagnostic findings were statistically significant (See Table S1).

### 3.5 Final Multivariable Model

The independent associations from the final model are summarized in Table 3. Items that retained a significant, independent association (*p* ≤ 0.05) with an increased likelihood of pain during RCT in the full model were patients being younger (OR = 1.374, *p* = 0.05), treatment (dental) fear (OR = 1.36, *p* = 0.03), mandibular tooth (OR = 1.50, *p* = 0.005), and number of diagnostic findings (OR = 1.18, *p* = 0.03 increase for each finding). In contrast, whether the dentist was an endodontist (OR = 0.64, *p* = 0.01) and the patient was non‐Hispanic White (OR = 0.60, *p* = 0.002) were both inversely associated with reported pain during RCT. Patients who reported severe pain prior to RCT were more likely to have pain during RCT, although the increased risk was not statistically significant in the final model (OR = 1.240, *p* = 0.13). Based on a pseudo R^2^, the model only explained about 9.5% of the variance in anaesthesia failure.

**TABLE 3:** Adjusted associations of dentist, patient and pre‐treatment clinical findings with whether the patient reported pain during Root Canal Treatment (RCT) of 3 or greater (on a zero [none] to 10 [as bad as can be] scale).

	Univariable^a^		Final^b^, model Pseudo *R* ^b^: 9.5%		
	Odds ratio	*p*	Odds ratio	95% confidence interval	*p*
*Dentist characteristic*					
Endodontist	0.61	0.01	0.64	0.46, 0.89	0.01
*Patient characteristics*					
Age: < 55 years	1.75	< 0.001	1.37	1.01, 1.86	0.05
Non‐Hispanic white	0.55	< 0.001	0.60	0.45, 0.81	0.002
Severe worst pain pre‐RCT	1.54	0.002	1.24	0.94, 1.66	0.13
Took pain meds^c^ for tooth	1.44	0.01	1.13	0.82, 1.56	0.5
RCT fear: Some to extremely afraid	1.62	< 0.001	1.36	1.05, 1.77	0.03
Stress 8 or greater (on 4–20 scale)	1.52	0.002	1.22	0.92, 1.62	0.17
*Pre‐RCT clinical findings*					
Mandibular tooth position	1.38	0.01	1.50	1.16, 1.93	0.005
Number of diagnostic findings^d^	1.29	< 0.001	1.18	1.02, 1.36	0.03

^a^ Adjusted for clustering of patients within dentist with generalized estimating equations (GEE); all characteristics with *p* < 0.1 in bivariate/domain models were included in a single regression.

^b^ All variables listed in the “univariable” column were included in a single multiple regression (the final model).

^c^ Medications, prescription or non‐prescription, taken during the 7 days prior to RCT.

^d^ Diagnostic findings: Tender to biting pressure, percussion, palpation, and cold.

### 3.6 Local Anaesthesia Techniques

Local anaesthesia administration techniques results are summarized in Table 4. The clinician administered additional local anaesthetic in 707 (41%) patients. The most common additional local anaesthetic was the same type as the first, given in the same area (*N* = 295; 42%), followed by the same type but in a different area (*N* = 239; 34%), intrapulpal injection (*N* = 206; 29%), periodontal ligament (PDL) space injection (*N* = 199; 28%), block anaesthesia different from first (*N* = 39; 6%), intraosseous injection not PDL (*N* = 25; 4%), and other (*N* = 37; 5%). The ‘other’ category was most commonly a second injection with a different local anaesthetic agent in the same location or a palatal injection. This was further broken down into provider‐type. When examined individually by type using a GEE, there was no difference in the use of additional local anaesthetics between endodontists and general dentists.

**TABLE 4:** Local anaesthesia techniques overall and by dentist type (general dentist or endodontist).

Local anaesthetic methods used	All		GD		Endodontist		*p* ^a^	Odds ratio
	*N*	%	*N*	%	*N*	%		
None listed	983	57%	454	58%	529	57%	0.7	0.96
Second injection same type and area	295	17%	144	18%	151	16%	0.2	0.86
Second injection same type/different area	239	14%	112	14%	127	14%	0.7	0.95
Block anaesthesia different from first	39	2%	16	2%	23	2%	0.6	1.22
Also needed: PDL injection	199	12%	73	9%	126	13%	0.006	1.53
Also needed: Intraosseous injection not PDL	25	1%	9	1%	16	2%	0.3	1.51
Also needed: Intra‐pulpal injection	206	12%	101	13%	105	11%	0.3	0.86
Also needed: Other	37	2%	7	1%	30	3%	< 0.001	3.70
Any add local anaesthetic (any except other above)	707	41%	318	40%	389	42%	0.6	1.05

Abbreviation: GD, general dentist.

^a^ *p*: from Chi‐square. Although certain techniques (e.g., PDL injection, intraosseous injection, and “other”) showed statistically significant differences in the bivariate analysis, none remained significant after adjustment for clustering of patients within dentist using generalized estimating equations (GEE).

Over half of those needing additional local anaesthesia required only one type (*N* = 473; 67%) while 162 patients (23%) required two types, and 72 patients (10%) required three or more types of anaesthesia. Patients reporting pain were more than twice as likely to have received additional local anaesthesia (OR = 2.32, *p* < 0.001).

## 4 Discussion

The patient‐centred definition of local anaesthesia failure in this study was based on a pain score of 3 or greater on a scale from zero to 10. In this study 274 patients met the anaesthesia‐failure definition, providing ~30 events per predictor in the final model. This definition resulted in a failure rate of 16%, which is similar to the 15% failure rate reported in the 2021 study by Weitz et al. (2021) that used a provider‐reported definition in a different pool of patients. In the Weitz et al. study, providers assessed the quality of local anaesthesia by selecting one of four options: excellent (patient felt nothing), adequate (patient experienced non‐painful sensations), marginal (patient experienced some pain), or less than marginal (patient experienced a lot of pain). Responses of “marginal” or “less than marginal,” were considered failures. Although our methods and those of Weitz et al. differ, both approaches reflect a similar threshold for defining failure, where pain beyond minimal is seen as clinically inadequate anaesthesia. In this study six independent predictors were identified: four risk factors (age < 55, dental treatment fear, mandibular tooth, and multiple abnormal diagnostic findings) and two protective factors (endodontist care and non‐Hispanic White ethnicity).

### 4.1 Psychosocial Factors

In this study, patients who reported being “somewhat to extremely afraid” before RCT were more likely to experience pain during the RCT procedure (OR = 1.48, *p* = 0.005). From an explanatory perspective, according to the gate control theory, psychological states can influence pain perception through descending central nervous system activity, meaning that pain experience is modulated not only by the peripheral stimulus but also by the patient's psychological state at the time of treatment (Melzack and Wall 1965). This partially aligns with PBRN evidence that fear and severe tooth pain co‐occur before RCT (Mungia et al. 2025b), although those findings were not independent after accounting for other pain‐related factors (Mungia et al. 2025a). Armfield (2010) revealed that dental fear is driven more by how patients perceive the dental visit (uncontrollable and dangerous) than by actual past painful experiences. Siegel et al. (2012) also documented that fearful patients tend to delay seeking care until pain is not tolerable, often presenting with more‐advanced disease. The self‐reinforcing nature of this cycle is supported by Laureano et al. (2023), who found that patients with recent dental pain had twice the prevalence of dental fear. This suggests that fear not only may directly influence the pain experience but also may contribute indirectly by causing patients to delay seeking care, resulting in more advanced disease and potentially, more difficult anaesthesia. A recent systematic review revealed that sedation drugs and audiovisual distractions might be useful for managing dental fear in adults (Lu et al. 2023).

Although dental treatment fear was a significant predictor of anaesthesia failure, other psychological constructs such as general anxiety, catastrophizing, and perceived stress were not retained in the final model. These findings suggest that dental fear, rather than generalized emotional distress, may be the most clinically relevant psychosocial factor in predicting anaesthesia outcomes during endodontic treatment. Given that fear is a modifiable predisposing factor, it is critical to implement clinical strategies aimed at reducing fear prior to treatment. Short‐acting anxiolytics or the use of nitrous oxide sedation can reduce physiologic arousal and improve patient comfort (Malamed 2010, 2019). An informed approach that includes an effective two‐way interaction, effective listening, and asking permission before initiating treatment steps, has been shown to improve trust and support a more cooperative clinical environment (Armfield and Heaton 2013). Additionally, screening for dental fear before treatment using a brief survey or Likert‐scale question, as employed in this study, allows the provider to identify at‐risk patients early and tailor the treatment plan accordingly.

### 4.2 Patient Demographic Factors

The observed racial and ethnic disparities in anaesthesia failure, specifically the significantly lower odds of reported pain among non‐Hispanic White patients during RCT (OR = 0.60, *p* = 0.002), may reflect structural inequities rather than biological differences. Minority populations are significantly more likely to reside in areas where dental health professional access is lower (Okunseri et al. 2008; Centers for Disease Control and Prevention 2024; American Dental Association Health Policy Institute 2022; Nasseh et al. 2023; Rahman et al. 2024). African American and Asian adults have been found to report significantly higher odds of oral pain compared to non‐Hispanic White adults, even after adjusting for socioeconomic and clinical variables (Aldosari et al. 2021). White adults are also more likely to have private dental insurance, compared to African American and Hispanic adults (Manski and Brown 2007). Although access to dental care and these related variables were not directly measured in the current study, the observed difference among different ethnicities warrants further investigation.

Our finding that patients younger than 55 years of age are more likely to report pain is consistent with findings from general pain and dental‐specific pain research, which show that younger adults tend to have greater pain sensitivity and report higher pain levels during procedures (Gibson and Helme 2001; Gibson and Farrell 2004; Lautenbacher et al. 2017). Physiologically, age‐related changes in the nervous system may reduce pain sensitivity in older adults. Studies show that peripheral and central nervous system processing of nociceptive stimuli diminishes with age, leading to decreased pain perception (Lautenbacher et al. 2017; Gibson and Farrell 2004). Psychosocial factors could also influence pain reporting in younger patients because they may have higher levels of dental anxiety, which could amplify pain perception (Klingberg and Broberg 2007).

### 4.3 Clinical Factors

In this study, the clinical factors significantly associated with local anaesthesia failure are well‐documented in the endodontic literature. Teeth presenting with multiple abnormal diagnostic findings, such as tenderness to percussion or biting and an abnormal cold test were more likely to be associated with patient‐reported pain during RCT (Nusstein et al. 2010). In such cases, local inflammation increases peripheral sensitization (Henry and Hargreaves 2007), which reduces the efficacy of local anaesthetics, and inflammatory mediators, including prostaglandins and cytokines, alter nerve excitability, making the tissues more resistant to anaesthesia (Byers and Närhi 1999; Hargreaves and Keiser 2002; Malamed 2019; Obadah et al. 2026). Although not statistically significant, patients who reported severe preoperative pain showed a trend toward greater pain during RCT (*p* < 0.1).

Prior studies involving patients presenting with moderate to severe preoperative pain have shown lower success rates with standard inferior alveolar nerve blocks, particularly in mandibular teeth diagnosed with irreversible pulpitis (Cohen et al. 1993; Nusstein et al. 1998; Drum et al. 2017; Dias‐Junior et al. 2021; Tortamano et al. 2009). A key limitation of the technique is its dependence on anatomical landmarks related to the innervation of the trigeminal nerve V3 compared to buccal or lingual infiltration. Mechanistic explanations for persistent pain in the presence of an apparently adequate mandibular block have also been proposed, including the central core theory and altered sodium channel physiology (e.g., TTX‐resistant channels) (Drum et al. 2017; Obadah et al. 2026). Together, these observations emphasize the importance of identifying patients at risk for local anaesthesia failure preoperatively. Those presenting with multiple abnormal diagnostic findings and severe preoperative pain may benefit from proactive anaesthetic strategies recommended in the endodontic literature (Nusstein et al. 2010).

### 4.4 Provider Factors

The present study indicates that patients treated by endodontists were less likely to experience significant pain during RCT, highlighting a notable protective effect associated with specialist care. This is consistent with the findings of Weitz et al. (2021), who reported that general dentists experienced nearly double the rate of anaesthesia failure in endodontic procedures compared to specialists. Several factors likely contribute to this difference. Endodontists receive advanced training in local anaesthesia, which can provide them with the ability to manage the challenges posed by inflamed pulpal tissues more effectively. In clinical practice, this training is reflected in their routine use of supplemental anaesthesia techniques, such as intraosseous and periodontal ligament injections, which have demonstrated higher success rates in symptomatic cases compared to standard blocks (Kanaa et al. 2012). Endodontists also confirm anaesthesia using objective methods such as cold testing, which provides a more accurate assessment of preoperative pulpal anaesthesia than soft tissue evaluation alone, which may justify providing additional anaesthetic before starting the procedure (Hsiao‐Wu et al. 2007).

Although general dentists and endodontists administered additional anaesthetics at similar rates (*p* > 0.05), patients treated by endodontists experienced significantly less pain. This suggests that the observed difference in patient‐reported pain may not reflect the amount of local anaesthetic administered, but rather the technical delivery of the drug. While our study was not designed to assess the use of supplemental anaesthesia as the primary outcome, these data reveal that patients who reported significant pain were over twice as likely to have received additional anaesthetic (OR = 2.32, *p* < 0.001). These findings emphasize that anaesthesia success depends not only on “how much” is given but also on “how” the drug is administered.

The 16% failure rate and the protective effect of endodontist care replicate findings from Weitz et al. (2021) who used a previous pool of patients from the network. The identification of dental fear as an independent psychosocial predictor extends that prior work and is novel in the practice‐based setting. The lack of association for TMD, catastrophizing, depression, and anxiety—after adjustment—suggests that fear related to dental situations may matter more than general emotional distress.

Several limitations should be acknowledged. First, the prospective observational design prevents causal inference. Second, anaesthesia failure was defined solely by patient‐centered outcomes. Third, data on specific anaesthetic agents (e.g., short vs. long‐acting anaesthesia) were not obtained. Finally, practice‐based research network studies still may introduce selection bias, as participating dentists may differ systematically from the broader dental population.

## 5 Conclusions

In the present study, the overall failure rate of local anaesthesia was 16%. Pre‐treatment fear was the only significant psychosocial predictor of intra‐operative pain. Mandibular teeth and those presenting with multiple abnormal diagnostic findings, such as cold sensitivity and percussion tenderness, were also more likely to be associated with anaesthesia failure. Additionally, younger patient age was significantly associated with increased pain reporting. From a provider perspective, patients treated by endodontists were significantly less likely to experience pain compared to those treated by general dentists. These findings support pre‐treatment fear screening and proactive anaesthetic strategies in patients presenting with multiple clinical risk factors.

## Author Contributions

L.J.L.: writing; R.O.‐Z.: review, editing; W.C.N.: review, editing; E.F.: statistical analysis, writing; A.S.L.: review, editing; E.W.N.L.: review, editing; G.H.G.: review, editing, funding; R.M.: review, editing; D.R.N.: review, editing.

## Funding

This work was supported by NIH grants U19‐DE‐22516 and U19‐DE‐28717. Opinions and assertions contained herein are those of the authors and are not to be construed as necessarily representing the views of the respective organizations or the National Institutes of Health.

## Ethics Statement

The PREDICT study was granted human research ethics approval by the University of Alabama at Birmingham (Protocol X161121003), the University of Minnesota (Protocol RNI00002290), and the University of Toronto (Protocol 34 105).

## Consent

All patients that participated in this study signed an informed consent.

## Conflicts of Interest

The authors declare is no conflicts of interest.

## Supporting information


**Table S1:** Models within each domain (broad category) of possible predictors of failed anaesthesia (patient reported pain during the RCT of 3 or greater on a zero [no pain] to 10 [as bad as can be] scale).

**Figure S1:** Flow diagram of included and excluded cases.

## Data Availability

The data that support the findings of this study are publicly available at the National Dental PBRN website at https://www.nationaldentalpbrn.org/recruiting‐ongoing‐upcoming‐completed/#1589296360026‐d0498e9a‐466e.
