# Supplementary material for: Clinical and Psychosocial Predisposing Factors Associated With Anaesthesia Failure During Non‐Surgical Root Canal Treatment: A National Dental Practice‐Based Research Network Study
Source: Int Endod J. 2026 May 18;59(9):1817–30. doi: 10.1111/iej.70171 (PMC13462444; doi:10.1111/iej.70171)
Supplement: Supplementary file 1 [file IEJ-59-1817-s002.docx]

**Supplementary Table 1. Models within each domain (broad category) of possible predictors of failed anesthesia (patient reported pain during the RCT of 3 or greater (on a zero [no pain] to 10 [as bad as can be] scale)**

|  |  | **Bivariate** | | **Joint** | |
| --- | --- | --- | --- | --- | --- |
| **Domain** | | **Odds Ratio** | **P^1^** | **Odds Ratio** | **P^2^** |
|  |  |  |  |  |  |
| Patient demographics | |  |  |  |  |
|  | Patient age: <55 years | 1.75 | <.001 | 1.59 | .005 |
|  | Patient: non-Hispanic white | 0.55 | <.001 | 0.61 | .002 |
|  | Has dental insurance | 1.34 | .048 | 1.28 | .13 |
| Pain sensitivity | |  |  |  |  |
|  | Current severe pain immediately prior to RCT | 1.58 | .006 | 1.33 | .1 |
|  | Worst pain 7 days prior to RCT was severe | 1.54 | .002 | 1.37 | .04 |
| Patient psychosocial scores | |  |  |  |  |
|  | RCT fear: Some to extremely afraid | 1.62 | <.001 | 1.51 | .003 |
|  | Anxiety score of 3 or higher (on a 2-8 scale) | 1.35 | .01 | 1.11 | .5 |
|  | Depression score of 3 or higher (on 2-8 scale) | 1.30 | .09 | 1.03 | .9 |
|  | Stress score of 8 or higher (on 4-20 scale) | 1.52 | .002 | 1.42 | .01 |
|  |  |  |  |  |  |
| Dentist pre-RCT assessment | |  |  |  |  |
|  | Mandibular | 1.38 | .01 | 1.39 | .01 |
|  | Number of diagnostic findings (0 to 4) | 1.29 | <.001 | 1.25 | <.001 |
|  | Swelling present | 1.47 | .07 | 1.26 | .2 |
|  | Curvature substantial or S-shaped | 1.38 | .095 | 1.33 | .14 |

^1^p: p-value adjusted only for clustering of patients within dentist with generalized estimating equations (GEE)

^2^p: p-value adjusted for all characteristics listed in the domain and for clustering of patients within dentist with GEE (only characteristics that retain a p<.1 were included in final model)
